# Socioeconomic Status and Hypertension among Teachers and Bankers in Addis Ababa, Ethiopia

**DOI:** 10.1155/2016/4143962

**Published:** 2016-05-22

**Authors:** Girma Fikadu, Seblewengel Lemma

**Affiliations:** Addis Continental Institute of Public Health, P.O. Box 26751/1000, Addis Ababa, Ethiopia

## Abstract

*Background*. The social and economic changes taking place in developing countries are influencing the pace at which hypertension and its risk factors are expanding. As opposed to the already established inverse association in developed nations, the association between socioeconomic status and hypertension in developing countries is poor and inconsistent. This study aims to determine the association between socioeconomic status and hypertension among teachers and bankers in Addis Ababa, Ethiopia.* Methods*. This study is based on a cross-sectional study conducted to assess the prevalence of NCDs in Addis Ababa, Ethiopia. The study was undertaken among workers of the Commercial Bank of Ethiopia and teachers of public schools in 2010.* Results*. Majority of participants were teachers (70.3%). Most of the respondents (54.1%) earn an annual income between 15,000 ETB and 48,000 ETB, and 51.9% of them have educational status of first degree and above. Among the socioeconomic factors income was strongly associated with the odds of having hypertension (AOR: 2.17 with 95% CI: 1.58–2.98).* Conclusions*. Higher burden of hypertension is observed among teachers and bankers in Addis Ababa, Ethiopia. Promotion of healthy behaviors and interventions that target higher income groups needs to be put in place.

## 1. Introduction

Recently, in addition to the high prevalence of infectious diseases, the incidence of noncommunicable disease is alarmingly increasing in the developing countries [1]. Although there are some more recent studies indicating the decline of CVD in Africa, NCDs are generally showing rapid rise in these countries [2]. One of the explanations for the increase is the economic and social transformation taking place in these countries, and the lifestyle changes resulted from such transformation [3].

Hypertension is one of the noncommunicable diseases that is advancing at fast pace in African countries [4]. The trend is unexpected given the socioeconomic status of people in these countries, as the disease is believed to be disease of the rich and developed nations [5].

Besides being a major cause of death, hypertension is a major risk factor for many chronic diseases like coronary heart disease, stroke, heart failure, kidney disease, and others [6]. It is responsible for about 6% (9 million) of deaths worldwide and affects about one billion people globally [5]. Hypertension is enormously affecting the working groups. Its global prevalence among older adults aged 25 and over is around 40% in 2008 [6]. The highest of this effect falls on the people in middle and low income countries [6]. Low income countries like Sub-Saharan Africa are experiencing unexpected rise in the incidence of hypertension [1]. The few studies conducted in Ethiopia are also showing high prevalence of the disease in the country. According to one study, 10.5% of the Ethiopian population has been estimated to have hypertension [7]. Another more recent study conducted in the capital shows that approximately 30% of adults in Addis Ababa have hypertension [8]. Added to the silent nature of the disease and the poor health seeking behavior of the people, as the country is going through a rapid economic development, and social and behavioral change, the figure is estimated to be higher.

Income, education, and occupation are the most commonly used indicators or measures of the socioeconomic status of an individual. Although its measurement is difficult in the developing countries, household income has shown consistent association to the general measures of health [9]. Educational status is also widely used as a measure of socioeconomic status and is related to many health outcomes [10]. Educational attainment reflects a household's ability to avoid risky behaviors and practice good health. Occupation is another common measure of socioeconomic status linking economic factors to health outcomes. It reflects health risks and protection factors related to the occupation and provision of source of income to practice good health behavior [11].

There are significant evidences indicating an inverse association between socioeconomic status and hypertension in the developed countries [12–14]. This is because in the developed countries individuals with high socioeconomic status may be the early adopters of healthy lifestyles that help to lower the risk of hypertension. But, in the developing countries, these groups are early adopters of harmful lifestyles characterized by smoking, diets with high energy and fats, and sedentary lifestyles [15]. The inverse association of socioeconomic status with hypertension is not evident in the developing countries.

The results from the few studies conducted in the developing countries have shown inconsistent results. For instance a study from Nigeria among adults aged 30–60 years found an inverse association between socioeconomic status and hypertension [16]. In contrary to this finding, a study on civil servants in the same nation found that those at higher occupational levels had higher hypertension when compared with those at lower occupational levels [15]. Another cross sectional home to home study conducted in urban population in Dar es Salaam, Tanzania, aged 25 to 64 came up with a result similar to that of the developed countries [17]. However, the use of different measurement of socioeconomic status and the stages of their economic development in these countries have made comparisons across other literatures difficult.

The results from many middle income countries are also showing positive association between socioeconomic status and hypertension. A study in rural Indian adults shows the highest socioeconomic group had almost double the prevalence of hypertension as those from the lowest socioeconomic group [18]. Another study within the same country has also showed a significantly positive relationship between socioeconomic status and hypertension [19]. In study undertaken among Jamaican women, the prevalence of hypertension is highest among the wealthiest women compared with the poorest [14].

There are significant study results indicating the high prevalence of hypertension among the bank workers [19, 20]. In contrast some studies undertaken in Africa are showing the prevalence of hypertension being higher among teachers compared to the bankers. In Ethiopia little is known about how socioeconomic factors may influence the distribution of hypertension. Therefore the current study aims to examine the association between socioeconomic status and hypertension among the bankers and teachers in the capital, Addis Ababa.

## 2. Materials and Methods

### 2.1. Study Design and Population

This study is based on a cross sectional study conducted for the purpose of assessing the prevalence of noncommunicable diseases among working adults in Addis Ababa, Ethiopia. The study was conducted by Addis Continental Institute of Public Health in collaboration with University of Washington Multidisciplinary International Research Training Program. Study populations are permanent employees of the Commercial Bank of Ethiopia and teachers in government schools in Addis Ababa.

A multistage probabilistic sampling strategy was used to identify and recruit participants [21]. First two study sites, the commercial bank of Ethiopia and public schools, were purposefully selected based on their work force stability. Then probability proportional to size sampling procedures was used to select both Commercial Bank of Ethiopia branch offices and government schools. From each of the selected locations, all employees were invited to participate. More details of the methodology are described elsewhere [21, 22].

### 2.2. Data Collection and Measurements

The study was conducted in accordance with the WHO's STEP wise approach for noncommunicable disease surveillance in the developing countries [21, 23]. Additional questions were also added to reflect better the context of Ethiopia [21].

### 2.3. Socioeconomic Status

Socioeconomic status is commonly conceptualized as the social class of an individual or group. It is often measured as a combination of education, income, and occupation [10, 12].

*Education*. Questions on completed education levels or grades were asked. The responses were grouped into two wider categories: diploma and below and degree and above. 

*Occupation*. It refers to the institutions in which the study populations are employed. 

*Income*. Three income categories are identified based on the 50% median income classification and used for analysis [24, 25].

*Blood Pressure*. It was measured using a digital measuring device (Microlife BP A50, Microlife AG, Switzerland) with the participant sitting after resting for at least 5 minutes. Blood pressure was measured 3 times, with at least 3 minutes between consecutive measurements. In accordance with the WHO recommendation the mean systolic and diastolic blood pressure from the second and third measurements were considered for analysis [26]. For the purpose of this study people with systolic blood pressure greater than or equal to 140 mmHg, or diastolic pressure greater than or equal to 90 mmHg, or people with normal blood pressure who are taking antihypertensive drug therapy were classified as hypertensive. Classification of hypertension was determined according to Joint National Committee on Prevention, Detection, Evaluation and Treatment of High Blood Pressure [27] (see Table 1).

*Weight and Height*. Participants were weighed using a solar-powered scale with an accuracy of ±100 grams. Their height was measured using an adjustable wooden measuring board, specifically designed to provide accurate measurements [22, 23]. 

*Body Mass Index*. Body mass index (BMI) was calculated as weight in kilograms divided by the square of height in meters. Classifications of BMI groups were done based on WHO recommendation. BMI < 18.5 represents underweight, 18.5 to <25 stands for normal weight, ≥25 to <30 refers to overweight, and obesity is decided when BMI ≥ 30 [28].

### 2.4. Statistical Analysis

SPSS (version 20.0) was used for statistical analysis. Data completeness and consistency were checked by running frequency on each variable. After excluding participants with missing BP measurement data and pregnant women (*n* = 339) from the original study sample, a total of 1866 study subjects were considered for the analysis. Frequencies and percentages were used for descriptive statistics to see the distribution of the different variables. Bivariate and multivariate logistic regressions were conducted to see the association between the measures of socioeconomic status and hypertension. Adjusted odds ratios (AOR) with 95% confidence interval were reported.

## 3. Results

### 3.1. Characteristics of the Study Population

A total of 51 schools and CBE's branch offices participated in the study. As the study has collected data on all the variables related to NCDs, for this study participants with no BP measurement data (*n* = 318) and pregnant women (*n* = 21) were excluded from the original study sample. Accordingly a total of 1866 study subjects (1124 men and 742 women) with complete data on all the variables were included in the study.

As Table 2 indicates, the majority, 1,124 (60.2%), of the participants were male and 742 (39.8%) were female with the male to female sex ratio of 1.5 : 1. The mean age of the participants was 36.03 with a standard deviation of 11.91 years. Table 2 shows the socioeconomic and demographic characteristics of the study population.

### 3.2. Socioeconomic Status

More than seventy percent of the participants were teachers by occupation. Majority of the respondents (54.1%) were in the middle income category earning between 15,000 and 48,000 Ethiopian birr per year. Slightly more than half (51.9%) of the participants had first degree and above and the other half (48.1%) are diploma and below, making educational status normally distributed (Table 3).

### 3.3. Lifestyle and Behavioral Risk Factors

As Table 3 indicates, the majority (55.3%) of the participants had a normal BMI, while 26.3% were overweight. The prevalence of obesity was 5.8% and underweight was 12.6%. 83 (4.4) of the participants are current smokers and 9.1% were previous smokers. 14.4% and 2.5% of the study participants are moderate and heavy drinkers, respectively. About 61% of the participants are not physically active. 4.8% of participants were diabetic and 13.9% of them have mental distress. Thirty percent of the participants have family history of hypertension.

The prevalence of hypertension in the study is 21% (95% CI = 19.15, 22.85). 127 (6.8%) of the hypertensive participants are currently receiving BP treatment. Only 278 (14.9%) of the participants are aware of their hypertension status before the interview.

**Figure 1:**
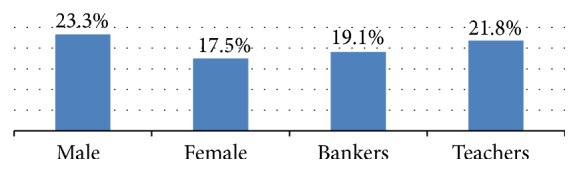
Prevalence of hypertension by sex and occupation among study participants in Addis Ababa, Ethiopia.

The results from the bivariate logistic regression indicate that males tend to be more hypertensive than females (OR: 1.43, 95% CI: 1.13–1.81) (Figure 1). The odds of hypertension increases with increase in age: 35–44 (OR: 3.11, 95% CI: 1.94–4.99), 44–55 (OR: 7.786, 95% CI: 5.021–12.075), and above 50 (OR: 14.23, 95% CI: 8.72–23.23).

A higher prevalence of hypertension was observed among middle income groups (OR: 2.26, 95% CI: 1.66–3.07); a statistically significant prevalence is also observed among the higher income groups (OR: 1.75, 95% CI: 1.21–2.52). However, as Table 4 shows, there was no statically significant difference in prevalence of hypertension among the educational categories (*P* value = 0.536) and occupation of the participants (*P* value = 0.197).

## 4. Discussion

This study identified hypertension as a significant health problem among bankers and teachers in Addis Ababa. 21% of the participants are found to be hypertensive. The study populations have showed a high prevalence of hypertension. The prevalence is 19.13% and 21.8% for bankers and teachers, respectively. These figures are higher compared with the 10.5% prevalence among the general population of Ethiopia [7] and lower than the 30% prevalence among adults in Addis Ababa in previous studies [8]. The prevalence of hypertension in this study is consistent with 19.3% reported in Nigeria [29] and 21.8% for Uganda [30].

All the significant factors which have proven to be related with the incidence of hypertension were entered into the multivariate regression model. In addition checks were conducted and no multicollinearity was found among the variables used for measuring socioeconomic status.

From the socioeconomic status measures used, only income has shown strong positive association with hypertension. Even after adjustment is made educational status and occupation have shown no significant association with the prevalence of hypertension.

The association of income with hypertension in this study is consistent with the findings from many low and middle income countries [1, 12, 31]. The middle and high income groups have shown higher odds of having hypertension. This finding indicates that in the context of the developing country having a higher income is not necessarily protective of health as it is in the developed world. This is probably due to the lifestyle of this group of people in these countries. These groups may use this income to provide more resources that may be used mostly for purchasing calorie-dense foods and excessive drinking, and in some instances it is a cause of sedentary lifestyles which are the underlying risk factors of hypertension.

Among this study population no significant association between education and hypertension was found. The absence of the association in this study may be due to the nature of the study population. In this study there is little variation in terms of educational status of the participants. However, studies done in the developing countries like Tanzania, Nigeria, and Ghana found out that education is inversely associated with the odds of developing hypertension [16, 31]. Similar findings were also documented in China [32], Iran [33], and Jamaica [14].

Occupation also has shown no statistically significant association with hypertension. Although there is a lack of studies that compared the two population groups, in studies conducted independently, the odds of developing hypertension is higher among the bank workers [19, 20]. In those studies it is explained that bankers' job is more stressful and sedentary (Table 5).

### 4.1. Additional Points

To the best of our knowledge this is the first study that assessed the association between socioeconomic status and hypertension in Ethiopia. The study adds to the scarce study results in this aspect. Even though the study has come up with important findings, there are certain limitations worth mentioning here. First of all, the fact that our study participants are all working professionals who are urban residents and fairly well educated makes the generalizing of the result to the larger population difficult.

Secondly as the study is based on secondary data the problem of social desirability bias is mentioned in the original study. Participants may withhold information regarding their life-style habits that may not be generally acceptable for working adults (smoking, drinking, etc.) which may result in an underestimation of these behaviors [34].

## 5. Conclusions

This study highlights the high prevalence of hypertension in the study population. As in many other developing countries hypertension is becoming a serious public health concern among working adults like teachers and bankers in Ethiopia. Like many developing and middle income countries, in this study better income is positively associated with higher odds of having hypertension. However, no association between hypertension and measures of socioeconomic status like education and occupation was found in this study.

## 6. Recommendation

As hypertension is becoming a serious public health concern in Ethiopia, it has to be given due concern in the health agenda of the country as one of top priority. Income does not play a direct role in increasing the odds of hypertension. It rather influences the practice of risky behavioral factors that are responsible for hypertension. Therefore promoting healthy lifestyles and interventions in lifestyle modifications related to the behavioral risk factors is recommended in reducing and controlling the prevalence of hypertension [27].

Awareness creation and promotion of healthy behaviors have to be widely conducted, especially in the better income groups so that they will make rational decision in choosing their behaviors. Particularly, the long term consequences that arise from these lifestyles have to be stressed to the society in general and these groups in particular.

Future studies are also highly recommended to confirm these findings in general population with varying measures of socioeconomic status.

## Figures and Tables

**Table 1:** 

High blood pressure (HBP)	Systolic blood pressure (mmHg)	Diastolic blood pressure (mmHg)
Normal	<120	<80
Prehypertension	120–139	or 80–89
Stage 1 hypertension	140–159	or 90–99
Stage 2 hypertension	≥160	or ≥100

**Table 2:** Distribution of socioeconomic and demographic characteristics among teachers and bankers in Addis Ababa, Ethiopia.

Characteristics	Frequency	Percent
Sex		
Male	1,124	60.2
Female	742	39.8
Mean age, years (±SD)	36.03 (11.91)	
Age (years)		
≤24	358	19.2
25–34	660	35.4
35–44	311	16.7
45–54	367	19.7
≥55	170	9.1
Religion		
Orthodox	1,455	78
Muslim	74	4
Protestant	284	15.2
Catholic	16	0.9
Others	37	2
Marital status		
Single	933	50
Married	813	43.6
Other	120	6.4
Occupation		
Bankers	554	29.7
Teachers	1,312	70.3
Educational status		
Diploma and below	897	48.1
Degree and above	969	51.9
Income		
Low (≤15000)	460	24.7
Medium (15001–48000)	1010	54.1
High (>48000)	396	21.2

**Table 3:** Distribution of behavioral risk factors among teachers and bankers in Addis Ababa, Ethiopia.

Characteristics	Frequency	Percent
BMI		
Underweight	235	12.6
Normal	1032	55.3
Overweight	490	26.3
Obese	109	5.8
Smoking status		
Nonsmoker	1614	86.5
Pervious smoker	169	9.1
Current smoker	83	4.4
Alcohol consumption		
Nondrinker	582	31.2
Low drinker	969	51.9
Moderate drinker	269	14.4
Heavy drinker	46	2.5
Physical exercise		
Yes	728	39
No	1138	61
Diabetes status		
Yes	90	4.8
No	1776	95.2
Mental distress status		
Yes	259	13.9
No	1607	86.1
Family history of hypertension		
Yes	563	30.2
No	1138	61
Not known	165	8.8

**Table 4:** Bivariate analysis for the association between socioeconomic status and hypertension among teachers and bankers in Addis Ababa, Ethiopia.

Variables	Hypertension		OR (95% CI)	*P* value
	Yes	No		
Sex				
Male	262	862	1.431 (1.132–1.809)	0.003
Female	130	612	1	
Age				
≤24	28	330	1	
25–34	60	600	1.179 (0.738–1.882)	0.492
35–44	65	246	3.114 (1.941–4.997)	<0.001
45–54	146	221	7.786 (5.021–12.075)	<0.001
≥55	93	77	14.235 (8.721–23.234)	<0.001
Income				
Low (≤15000)	59	401	1	
Medium (15001–48000)	252	758	2.260 (1.660–3.075)	<0.001
High (>48000)	81	315	1.748 (1.211–2.521)	0.003
Education				
Diploma and below	183	714	0.932 (0.746–1.165)	0.536
Degree and above	209	760	1	
Occupation				
Bank workers	106	448	1.178 (0.918–1.511)	0.197
Teachers	286	1026	1	

**Table 5:** Multivariate logistic regression analysis: association between socioeconomic status and hypertension among teachers and bankers in Addis Ababa, Ethiopia.

Variables	Adjusted OR	95% CI for AOR	*P* value
Income			
Low (≤15000)	1		
Medium (15001–48000)	2.168	1.579–2.978	<0.001
High (>48000)	1.955	1.312–2.913	0.001
Education			
Diploma and below	1.042	0.825–1.317	0.728
Degree and above	1		
Occupation			
Bank workers	0.750	0.569–0.989	0.042
Teachers	1		
